# Supplementary material for: Evidence of microplastics in water and commercial fish from a high-altitude mountain lake (Lake Titicaca)
Source: PeerJ. 2022 Nov 9;10:e14112. doi: 10.7717/peerj.14112 (PMC9653051; doi:10.7717/peerj.14112)
Supplement: Supplemental Information 2 [file peerj-10-14112-s002.docx]

| Species | Landing zones | N | Habitat | Diet | TL (mm) ± SD; range (mm) | W (g) ± SD; range (g) | Source for habitat, feeding habits and diet |
| --- | --- | --- | --- | --- | --- | --- | --- |
| *Orestias agassizii* | Cachilaya, Huarina and Desaguadero | 407 | Ubiquitous (littoral, benthic and pelagic) | Omnivorous: Amphipods, mollusks, insects and plankton | 114 ± 25; 62-203 | 27 ± 18; 5-116 | (Lauzanne, 1982, 1992; Parenti, 1984; Vila, Pardo & Scott, 2007; Maldonado et al., 2009; Ibañez et al., 2014; Monroy et al., 2014; Loayza et al., 2020) |
| *Orestias luteus* | Cachilaya, Huarina and Desaguadero | 347 | Benthic | Omnivorous-Microcarnivorous: Amphipods, mollusks, insects, eggs | 108 ± 18; 66-172 | 30 ± 16; 4-92 | (Lauzanne, 1982, 1992; Parenti, 1984; Vila, Pardo & Scott, 2007; Maldonado et al., 2009; Ibañez et al., 2014; Monroy et al., 2014; Loayza et al., 2020) |
| *Trichomycterus dispar* | Cachilaya and Huarina | 287 | littoral, benthic | Omnivorous-Microcarnivorous: Amphipods, mollusks, insects, and eggs | 139 ± 15; 109-193 | 26 ± 10; 12-80 | (ALT, 2003; Ibañez et al., 2014) |
| *Odontesthes bonariensis* | Cachilaya, Huarina and Desaguadero | 242 | Pelagic, littoral | Carnivorous-Piscivorous: Amphipods, zooplankton, insects, fish and frogs | 213 ± 55; 136-370 | 70 ± 63; 14-330 | (Loubens, 1989; Vila, Pardo & Scott, 2007; Monroy et al., 2014) |
